# Renal failure during chemotherapy: renal biopsy for assessing subacute nephrotoxicity of pemetrexed

**DOI:** 10.1186/s12885-017-3705-7

**Published:** 2017-11-16

**Authors:** Maureen Assayag, Philippe Rouvier, Marion Gauthier, Ghania Costel, Philippe Cluzel, Lucile Mercadal, Gilbert Deray, Corinne Isnard Bagnis

**Affiliations:** 1 Pitié Salpêtrière Hospital, 75013 Paris, France; 2 Montargis Dialysis Center, Amilly, France

## Abstract

**Background:**

Pemetrexed, a multitargeted antifolate cytotoxic agent, is currently used primarily in combination with cisplatin for metastatic non-small cell lung cancer and for malignant mesothelioma. Acute renal toxicity of pemetrexed has been recently described with polychemotherapy, in which the individual responsibility of each drug is difficult to establish. Only one recent report documents renal involvement in long-term exposed patients.

**Case presentation:**

We report on a case of rapidly progressive nephropathy leading to the cessation of platinum salts and the secondary interruption of pemetrexed and bevacizumab. Acute tubular necrosis shown on the renal biopsy could potentially be due to pemetrexed. Persistent severe renal failure after the resumption of all drugs led to new treatment lines with gemcitabine (while the glomerular filtration rate was below 30 ml/min/1.73m^2^), then followed by Taxol.

**Conclusions:**

The optimal strategy with regard to renal complications in cancer patients is not clear. Acute or chronic loss in renal function generally leads to a new treatment line, possibly impairing the overall success of the treatment. The use of chemotherapy in patients with a glomerular filtration rate below 30 ml/min/1.73m^2^ is usually associated with an increased risk of side effects when not contraindicated by renal elimination of the drug.

## Background

Most cancer patients receive polychemotherapy. The anticipated efficacy may only be attained, however, if the regular protocol is conducted in its entirety. Renal failure usually leads to delayed or interrupted chemotherapy, and in the absence of full treatment, the expected benefits may be elusive. Alternatively, second- or third-line treatment is offered if drugs are manageable with the maintenance of a low glomerular filtration rate (GFR).

As shown in several studies [1], renal failure appears therefore to be one of the main causes for chemotherapy discontinuation. Indeed, individual compliance with the use of drugs in polychemotherapy regimens is usually difficult to confirm. The reversibility of acute renal events determines whether a strategy can be followed, since many drugs cannot be prescribed in case of severe renal failure. Prevention measures, rapid diagnoses and extensive work-ups to determine the causes of GFR decline are necessary and are urgently required before stage 4 or 5 occurs, in which case irreversibility would prevent any future cancer therapy.

Anticancer drugs may exhibit different forms of renal toxicity (namely tubular, interstitial, glomerular or vascular), and often only renal biopsy can shed light on the mechanism of renal function decline. We describe a typical case of a cancer patient treated with 3 different drugs exhibiting severe renal failure. We emphasize the therapeutic changes that were necessary due to non-reversible renal failure and question the potential consequences on the outcome. We also discuss the benefits of renal biopsy in helping to diagnose drug-induced renal toxicity.

## Case presentation

A 66-year-old woman was admitted in March 2015 to Pitié Salpêtrière Hospital for rapidly progressive kidney failure. TNM stage IV lung adenocarcinoma had been diagnosed in December 2013 with pleural and nodal metastasis. Treatment began in January 2014 with three sessions of external radiotherapy and six sessions of chemotherapy with cisplatin (75 mg/m^2^), pemetrexed (500 mg/m^2^) and bevacizumab (10 mg/kg). Her only other past medical history was dyslipidemia. She had no diabetes and no hypertension. Before beginning chemotherapy, her plasma creatinine level was 50 μmol/L, corresponding to an estimated GFR (eGFR) of 113 ml/min/1.73m^2^ using the simplified Modification of Diet in Renal Disease (MDRD) equation.

In April 2014, the plasma creatinine level rose to 92 μmol/L. The medical team first suspected Cisplatin nephrotoxicity, therefore the chemotherapy protocol was changed, and only Pemetrexed and Bevacizumab were maintained from June 2014 at the same doses. However, the kidney function continued to worsen, with a creatinine plasma level of 111 μmol/L in August, and 154 μmol/L in November, corresponding to an eGFR of 42 ml/min/1.73m^2^. Chemotherapy sessions were cancelled in December 2014, earlier than expected, when the creatinine plasma level rose to 196 μmol/L, corresponding to an eGFR of 24 ml/min/1.73 m^2^, which then became a contraindication to treatment. At this time, there was no hypertension, proteinuria, hematuria or leukocyturia detected by urine dipstick.

After discontinuation, the creatinine plasma level continued to rise, to 266 μmol/L in January and 297 μmol/L in February 2015. No other treatment was given at this time, especially no non-steroidal anti-inflammatory drugs (NSAIDS) or antibiotics.

**Fig. 1:**
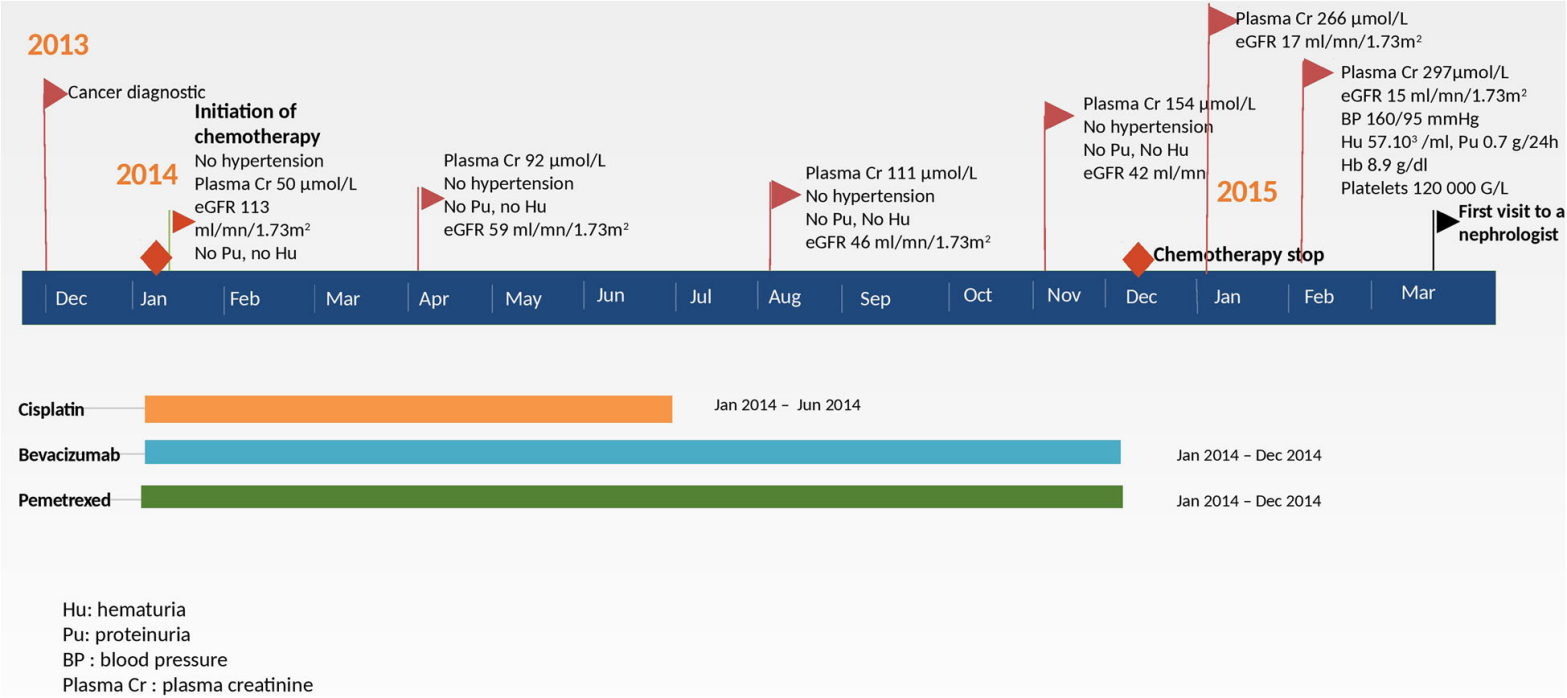
Timeline of patient’s renal function during chemotherapy

In February 2015, the patient developed hypertension with no extra-renal clinical signs, and was referred to a nephrologist. The 24-h proteinuria was estimated at 0.7 g. Microscopic hematuria appeared (57.10^3^/ml), without leukocyturia. Blood tests showed thrombopenia (120.10^3^ G/L) and anemia (8.9 g/dL), with no laboratory signs of hemolysis or schizocytes (Fig. 1). Antineutrophil cytoplasmic antibodies were absent, as were antinuclear and anti-DNA antibodies. There was no protein activation associated with complement. No gamma globulin abnormalities were seen in plasma protein electrophoresis. The kidney ultrasound showed two kidneys of normal size and shape, well differentiated, with a regular outline and no impediments.

**Fig. 2:**
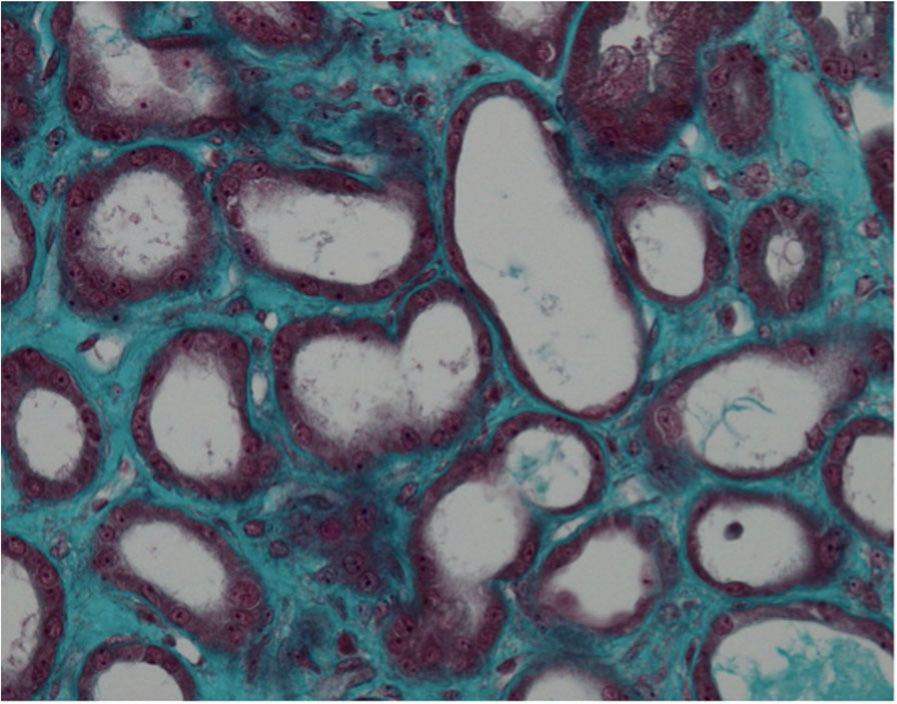
Kidney biopsy photographs; acute tubular necrosis with tubular cell necrosis and brush border loss. Glomeruli and interstitial compartment are normal. Immunofluorescence staining was negative

**Fig. 3:**
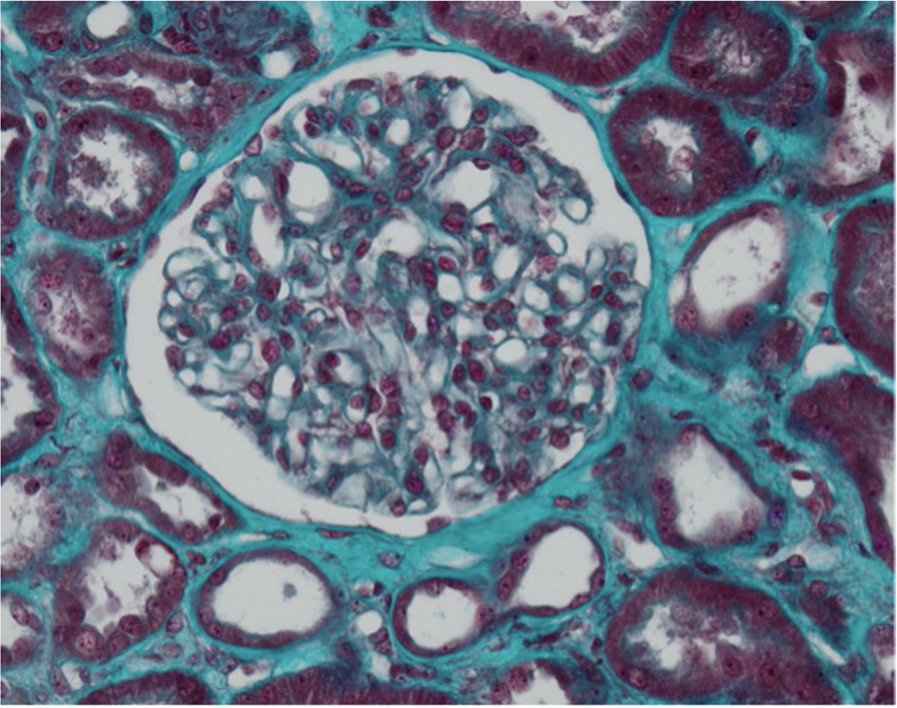
Kidney biopsy photographs; acute tubular necrosis with tubular cell necrosis and brush border loss. Glomeruli and interstitial compartment are normal. Immunofluorescence staining was negative

The kidney biopsy, shown in Figs. 2 and 3, revealed acute tubular necrosis. The glomeruli, interstitial compartment and vessels were normal. There were no thrombotic microangiopathic lesions, glomerular or tubular basement membrane deposits, or arteriolar hyalinosis. Immunofluorescence did not show any immune complex deposits. Electron microscopy was not performed.

The renal function did not recover. The patient was discharged and then followed by her medical team closer to home. In December 2015, she had a plasma creatinine level of 266 μmol/L. The GFR remained later low (around 17 ml/min/1.73m^2^) but with no need for dialysis until December 2016.

The anticancer treatment was prolonged in 2015 with 5 cycles of gemcitabine, and a four-month treatment-free period. In 2016, she received 6 cycles of gemcitabine and 6 cycles of paclitaxel. The lung CT scan was stable at the last visit.

## Discussion

This case illustrates rapidly progressing acute renal failure documented by acute tubular necrosis in a patient treated with cisplatin, bevacizumab and pemetrexed.

Cisplatin, a widely used chemotherapeutic agent for lung cancer, has the well-known side effect of nephrotoxicity. Almost 20% of patients receiving high-dose cisplatin develop acute kidney injury (AKI). One-third of AKI cases occur within a few days of administration, despite appropriate preventive measures (i.e. adequate hydration, dose adjustments) [2, 3]. Cisplatin has been traditionally combined with gemcitabine for metastatic small cell lung cancer. In more recent protocols, a switch from gemcitabine to pemetrexed, an antifolate cytotoxic agent with lower hematologic toxicity [4], has been used. Pemetrexed is a multitargeted antifolate agent with demonstrated efficacy in pleural mesothelioma and metastatic lung adenocarcinoma [5]. Of note is that it is the first agent to induce improvement in the prognosis of malignant pleural mesothelioma [6]. Pemetrexed inhibits several folate-dependant enzymes involved in purine and pyrimidine synthesis and thus, DNA synthesis. Its main toxicity is linked to vitamin deficiencies (folate and B12) and ranges from nausea/vomiting and diarrhea to skin rash, mucositis and neutropenia [7]. Pemetrexed is almost exclusively eliminated in the urine. Accumulation may occur in case of pleural or peritoneal effusion, and cumulative side effects may appear. Dialysis does not seem to be efficient for eliminating pemetrexed [8].

As with cisplatin, pemetrexed causes tubular toxicity, whereas gemcitabin and bevacizumab induce vascular defects [9, 10]. This toxicity seems to appear despite nephroprotective measures. Tubular cell apoptosis seems to be the common feature of pemetrexed toxicity, probably secondary to its accumulation and folate metabolism blockade. One case of nephrogenic diabetes insipidus appearing with pemetrexed has also been reported [11].

The effects of bevacizumab are different. A monoclonal antibody that binds to vascular endothelial growth factor (VEGF) receptors, thus inhibiting its angiogenic effects [12], it is known to induce glomerular changes responsible for proteinuria and some microangiopathy. Tubular injury is very rarely reported with bevacizumab nephrotoxicity but can be seen histologically. Indeed, one article reports five cases of AKI in patients treated with vascular endothelial growth factor inhibitors (four with bevacizumab and one with sorafenib) and highlights the fact that tubular injury with focal necrosis had been seen in all five cases, possibly in addition to thrombotic microangiopathy lesions [13, 14].

An increasing number of cases documenting acute kidney insult with pemetrexed have been published [15, 16]. In every case the authors describe an acute profile of kidney function degradation occurring 1 or 2 weeks after treatment initiation, with further degradation after stopping therapy [17]. In a recent study, around 50% of patients with maintenance treatment combining pemetrexed and bevacizumab had to stop due to acute kidney injury [1].

If renal toxicity occurs, the causative agent may be difficult to identify. Our case report illustrates the situation of many patients undergoing polychemotherapy treatment. In this case of acute kidney injury, the absence of thrombotic microangiopathy did not support the role of bevacizumab, but the mild proteinuria might have. Cisplatin could have been responsible, since the renal biopsy showed acute tubular necrosis, which is the most frequent elementary histological lesion expected in cisplatin toxicity; however its reduction or total discontinuation usually allows renal recovery in a few weeks [2, 3, 18]. The deterioration of renal function, despite the elimination of cisplatin months before, tends to rule out the responsibility of platinum salts in our case. Pemetrexed could be the causative agent here, as the renal histology is similar to that seen in other reported cases, and due to the timing and the non-resolution after discontinuation.

If there is no evidence for the cause of acute kidney injury in cases of polychemotherapy, we believe that renal biopsy can help by describing typical, or less typical, lesions. Some types of lesions have been attributed to certain types of chemotherapies (Table 2).

Most articles describe the expected or possible renal side effects during chemotherapy [19]. We suggest that appropriate renal monitoring could allow for early intervention in reducing the consequences of renal toxicity on further treatment (Table 1). Optimal monitoring includes a baseline evaluation to detect patients with preexisting nephropathy and patients at high risk of renal impairment. A few simple questions should be asked at the treatment initiation: is there a history of a renal failure, hematuria or proteinuria? Should we adapt monitoring to this particular patient because of an individual risk score higher than others? Should his/her usual medication be adjusted? Is the patient usually exposed to nephrotoxic drugs (NSAIDs, lithium, etc.) besides cancer treatment?

**Table 1:** Optimal monitoring and care of renal function during chemotherapy [20]

	Before treatment	During treatment	In case of renal impairment
Clinical	Evaluation of renal *risk*: - Familial renal history - Personal renal history (renal stones, recurrent cystitis, renal surgery, chronic kidney disease, acute renal failure) - Comorbidities associated with renal impairment (e.g. diabetes, hypertension) - Combined therapy associated with increased renal risk (all nephrotoxic drugs, e.g. NSAIDS, lithium)	- Patient education (for ambulatory treatment): -Home monitoring of weight and blood pressure -In case of vomiting/diarrhea with a significant weight loss (5%), patients should call their center -In case of vomiting/diarrhea with a significant weight loss (5%), diuretics and/or ACE inhibitors or ARBs should be adjusted/stopped for a few days (call the center) - Estimation of the severity of side effects (vomiting, nausea, anorexia, fever) - Quantification of dehydration (weight loss) - Blood pressure control and screening for orthostatic hypotension - Choose appropriate imaging strategy (prefer imaging with no contrast media whenever possible) and prior hydration	- Discuss hospitalization - Evaluate hydration status (e.g. edema, blood pressure, thirst, skin dryness) - Blood pressure - Bleeding or hematomas, cutaneous vasculitis, - Quantify urinary volume (oliguria) - Estimate clinical need for dialysis (pulmonary edema, hyper-hydration) - Preserve non-dominant arm venous network for potential need for arteriovenous fistula - Avoid subclavicular intravenous catheterizations (high risk for proximal venous thrombosis/stenosis and loss of chance for arteriovenous access) - Try avoiding urethral catheter (to decrease risk of urinary tract infection) - Stop every unnecessary drug and/or adjust dosage
Laboratory	- Best estimation of glomerular filtration rate (usually with sMDRD formula) or specific formula (such as Calvert’s formula for platinum prescription [21]) - Baseline hemoglobin, platelets, LDH and haptoglobin (allowing comparison with future abnormalities) - Urine dipstick (leukocytes, hematuria, proteinuria, glycosuria) and protein- creatinine ratio and identification of the origin (tubular or glomerular)	- Best estimate glomerular filtration rate and compare to previous values (+ urea value) - In case of poor creatinine value due to rapid changes in muscle mass or severe malnutrition, 24-h creatinine clearance gives appropriate results - Hemoglobin, platelets, LDH, haptoglobin, schizocytes, albuminemia - Urine dipstick for hematuria, proteinuria, leukocyturia In most cases, perform before and 8 to 10 days after each chemotherapy session and every month. Usually discuss next results and adjust strategy depending on latest results	- Estimate GFR and metabolic complications of GFR decrease in acute cases (hyperkalemia, acidosis, hyperphosphataemia, hyperphosphatemia, hypocalcemia, hypomagnesemia) - Plasma hemoglobin (and iron stores) - 24-h proteinuria and qualitative assessment of urinary proteins - Urinary ions and urine- plasma ratio for sodium, urea, and fractional excretion of sodium. - LDH, haptoglobin, schizocytes, albuminemia

It is particularly important to monitor clinical and laboratory renal parameters during treatment. For example, significant loss of weight from one cycle to the other (more than 5% of the initial weight), digestive disorders (diarrhea, vomiting) or hypotension are markers of dehydration and subsequently of the risk of functional renal failure. If creatinine increases, the first questions to be asked are: Is it pre-renal failure due to dehydration? Should I modify the symptomatic treatment? Or start rehydration? Are there factors supporting organic renal failure such as proteinuria, hematuria, leukocyturia, anemia or hemolysis? If the answer to the last question is yes, the chemotherapy should be suspended and the patient immediately referred to a nephrologist.

Being closer to the clinical situation, from a clinical and pragmatic viewpoint, we also share a strategy of action in case of early renal abnormality during treatment (Table 2). Randomized protocols define the usual timelines for laboratory monitoring based on experience with low-risk patients. In “real life”, cancer patients may indeed be at very high renal risk. Closer laboratory monitoring may allow a toxic drug to be resumed earlier and the initiation of an early treatment (steroids, plasma exchanges, dialysis, etc.). The list of markers that should be monitored may be tailored depending on the drugs received (glycosuria if Fanconi tubulopathy is expected, for example). In any case, drug dosage adjustment is mandatory if renal function declines. In the event of rapidly progressive renal impairment, hospitalization is highly recommended.

**Table 2:** Importance of renal biopsy in cancer patients

Orientation	Clinical situation	Importance of renal biopsy	Drugs involved
Prerenal failure	Weigh loss, dizziness, skin dryness, low blood pressure, tachycardia, oliguria.	No biopsy	All drugs associated with dehydration, vomiting or diarrhea
Obstructive renal failure	Pelvic cancer localization Suspicion or demonstration of ureteral dilation No ureteral stenting in presence of peritoneal carcinosis Oliguria, anuria Hypogastric pain or in the lumbar fossa Hematuria with clots	No biopsy	All drugs inducing a lysis syndrome or renal stones
Glomerular disease	Hypertension, edema Proteinuria composed mostly with albumin, hematuria without clots Nephrotic syndrome (plasma albumin below 30 g/L and nephrotic range proteinuria)	Perform immediate renal biopsy	- Interferon: podocytopathy inducing clinically minimal change disease, focal segmental glomerulosclerosis [22] - Anti-VEGF agents: hypertrophy of glomerular endothelial cells, widening of the subendothelial space [23, 24] - Tyrosine kinase inhibitors: Gefitinib: minimal changes [25, 26] Sorafenib [27] - Anthracyclines: Doxorubicin: focal segmental glomerulosclerosis [28]
Tubular disease	Proteinuria composed mostly with low weight proteins Leukocyturia No hematuria	Perform delayed renal biopsy if - If no resolution after 2 to 4 weeks - If clinical and biological picture is not clearly in favor of tubular disease	- Antimetabolites: Methotrexate: precipitation on renal tubules and tubule cells apoptosis [29] Pemetrexed: renal tubular cells apoptosis - Alkylating agents: Platinum salts: renal tubular cells apoptosis [3, 18] Ifosfamide: acute proximal tubular injury inducing Fanconi syndrome, diabetes insipidus [30] - Hormone therapy: Androgen deprivation therapy: acute tubular injury [31] - Anti-EGFR agents: Cetuximab: hypomagnesemia due to magnesium transport channel alteration in the loop of Henle [32] - mTOR inhibitors: Everolimus: antiproliferative effects and induction of tubular cells autophagy [33]
Interstitial disease	Cutaneous rash, liver enzymes increase, hypereosinophilia, urinary eosinophils	Essential and with no delay	- Acute interstitial disease: bevacizumab [34, 35], sunitinib [36, 37, 38], ifosfamide [39, 40], carboplatin [41], gemcitabin [38], methotrexate [38], interferon [42], Bcg therapy [43–45] - Chronic interstitial disease [24]: ifosfamide, carboplatin, doxorubicin
Vascular disease	Presence of schizocytes, drop in platelet count, undetectable haptoglobin or low level in an inflammatory context Cutaneous vasculitis	Perform biopsy if platelet level compatible	VEGF inhibitors: bevacizumab [46, 47], sorafenib [48], gemcitabine [12, 13, 49, 50], sirolimus [51], afilbercept [52, 47] Antitumor antibiotic: mitomycin C [53] Interferon [54] Tyrosine kinase inhibitors: Imatinib [55, 47]

## Conclusion

In conclusion, we would like to highlight the importance of close renal function monitoring in cancer patients. Anticipating side effects through clinical evaluation, laboratory tests and early discussion of renal biopsy may positively influence the outcome of cancer disease by providing the best chances for therapy completion.

## Abbreviations

ACE: Angiotensin-converting enzyme
AKI: Acute kidney injury
ARBs: Angiotensin II receptor blockers
eGFR: Estimated glomerular filtration rate
GFR: Glomerular filtration rate
MDRD: Modification of diet in renal disease
NSAIDS: Nonsteroidal anti-inflammatory drugs
TNM: Tumor, node, metastasis (classification of malignant tumors)
VEGF: Vascular endothelial growth factor
